# Inborn mechanisms of food preference and avoidance: the role of polymorphisms in neuromodulatory systems

**DOI:** 10.3389/fnmol.2013.00016

**Published:** 2013-07-05

**Authors:** Carmelo M. Vicario

**Affiliations:** School of Psychology, University of QueenslandBrisbane, QLD, Australia

In a recent issue of Current Biology, Ventura and Worobey (2013) reviewed the literature about the variables which affect the development of food preferences across the lifespan.

Among the numerous factors discussed by the authors the genetic influence seems to cover a key role.

The review addressed this aspect by discussing the growing research about the genetic factors which are responsible of the individual differences in perceiving flavors. For example, the authors reported that the TAS2R38 gene polymorphism is predictive of the level of bitterness (strong, moderate, or absent) perceived when tasting some particular compound (i.e., the phenylthiocarbamide and propylthiouracil—Bufe et al., 2005).

These references, however, do not clarify which factors might influence the subjective dislike and/or preference associated to a specific compound. In fact, although bitterness may reduce the food consumption in both of adult and children (Bell and Tepper, 2006; Dinehart et al., 2006), there is no direct relationship between bitterness and food dislike. The same authors observe that data for an association between bitter sensitivity and preferences remain equivocal, as the literature provides contrasting results (e.g., Keller et al., 2002; Dinehart et al., 2006; Jerzsa-Latta et al., 1990; Anliker et al., 1991) about the relationship between the sensitivity for bitter flavor and preferences for the consumption bitter foods.

Therefore, what Ventura and Worobey (2013) discussed is how the polymorphism of some genes might be predictive of the intensity in perceiving a taste (i.e., bitterness), though there is no mention of the genes involved in the encoding of the hedonic/disliking experience in feeding specific compounds.

Nevertheless, other genetic variables, which affect the disgust sensitivity, the perceived palatability of food, and the motivational state in feeding, might synergistically contribute in influencing the process of taste perception and preference. In particular, one might refer to the genetic mechanisms involved in the expression of neuromodulators such as the dopamine, serotonin, and opioids.

Dopamine and serotonin seem principally implicated in the regulation of incentive/motivational states of feeding (food “waiting”). The opioids have a role in the regulation of mechanisms of the hedonic impact (food “liking”), although they are able to modulate the “waiting” processes as well (Cools et al., 2011). Some recent work suggested that both “waiting” and “liking” might critically contribute in taste perception and preference (Peciña and Smith, 2010).

The research on people affected by eating disorders such as Anorexia Nervosa (AN) and hyperphagia, which are characterized by two antithetic feeding behaviors, provide important evidence in support of the neuromodulator hypothesis.

The core of the AN disorder is characterized by a marked disgust sensitivity for food and the human body (Troop et al., 2000; Harvey et al., 2002). Dopaminergic alterations, particularly in striatal circuits, seem to be responsible of a deranged reward and decreased food ingestion in this clinical population (Kaye et al., 2009). The reduced food ingestion of AN has been associated even to an increased serotonin 5-HT neurotransmission (Steiger, 2004).

From a genetic point of view, there is evidence that the expression of the D2 dopamine receptor might play a role in the feeding behavior of AN. Bulik et al. (2005) have shown that genetically transmitted variation in the expression of the D2 dopamine receptor, mediated by functional polymorphisms affecting its transcription and translation efficiency, may make people vulnerable to AN conducts.

Serotonin polymorphism has been also associated to AN. A recent meta-analysis (Calati et al., 2011) recognized the 5-HTTLPR S allele as a factor of high risk for AN. Moreover, the study of Brown et al. (2007) reported that the polymorphism in the serotonin 1D (HTR1D) receptor gene might determine AN. Finally, the opioid delta receptor OPRD1 might cover a role in the aetiology of AN. This is supported by the evidence that the polymorphism in OPRD1 receptor genes is significantly associated to restricting AN (Brown et al., 2007).

Hyperphagia is characterized by a strong desire to consume palatable, high calorie foods. Disgust seems to be reduced in these patients, which could, at least partly, explain their increased appetite (Houben and Havermans, 2012). In support of their suggestion, Houben and Havermans (2012) documented that women with a higher Body Mass Index showed decreased core disgust and contamination disgust. Thus, they appear to have a higher threshold for rejecting food products, which may explain their predisposition to overeat.

It was already shown that a reduced serotonin 5-HT patter of neurotransmission may precipitate in compulsive eating behavior (Blundell, 1986). Moreover, it has been suggested that obese individuals overeat in order to compensate for deficits in the dopaminergic reward system (Hardman et al., 2012). In fact, obese adults exhibit a deficiency in dopaminergic signaling in the striatum, as they have diminished availability of the D_2_ receptor in this region compared to lean individuals (Wang et al., 2001).

Even for this clinical condition, interesting insights are provided by the literature on the neuromodulators polymorphism. For example, a recent study (Dykens et al., 2011) has shown that Prader–Willi syndrome genotyped for the tryptophan hydroxylase 2 (TPH2) G703-T polymorphism had significantly high hyperphagic behavior. The polymorphisms in TPH2 are implicated in the rate-limiting enzyme in the biosynthesis of serotonin in the brain.

There is also evidence of a relationship between dopaminergic polymorphism and hypherphagia. For example, a strong relationship was shown between a gene involved in the synthesis of dopamine, the A1 allele of the DRD2/ANKK1-TaqIA, and obesity (Felsted et al., 2010). The recent study of Felsted et al. (2010) also demonstrated a specific association between the TaqIA A1 polymorphism, which is associated with diminished dopamine D(2) receptor density (also involved in higher body mass, and food reinforcement) and the brain response during ingestion of a palatable food.

Finally, it was shown (Davis et al., 2009) that the obesity associated to the Binge Eating Disorder (BED) is related to the A118G polymorphism of the mu-opioid receptor (OPRM1) gene. This suggests that the proneness to binge eating may be influenced by a hyper-reactivity to the hedonic properties of food.

The evidence that the polymorphism of the above mentioned neuromodulators is able to affect the feeding behavior by influencing the hedonic experience and the motivational state of the individual, certainly provide intriguing elements of investigation for disclosing which factors might have an early influence on taste perception and preferences. In this framework of reference, it might be intriguing also to explore if the mentioned polymorphisms influence taste receptor function and the perceived palatability of food. This might be of particular relevance for serotonin, which is already known to participate within the bud (Kaya et al., 2004; Huang et al., 2005). For example, in the study of Kaya et al. (2004) it was shown that 5-HT (1 A) and 5-HT (3) receptors are expressed in taste buds. In particular, immunocytochemistry analysis with a 5-HT (1 A)-specific antibody demonstrated that subsets of taste receptor cells were immunopositive for 5-HT (1 A).

However, we should keep in mind the study of de Araujo et al. (2008) which reported that postingestive effects can influence food preferences independently of palatability, via reward circuits' modulation.

This supports the suggestion that the inborn taste preference and avoidance might be founded on mechanisms of molecular polymorphism involving a central regulation; while the molecular polymorphism affecting the taste receptors might express its role by regulating the intensity of the hedonic/ disliking experience associated to a flavor.
